# Comparison of Pre‐ and Postoperative Gut Microbiota Diversity in Patients With Rectal Cancer Undergoing Stoma Creation and Closure

**DOI:** 10.1002/ags3.70094

**Published:** 2025-09-16

**Authors:** Yusuke Suzuki, Wataru Osumi, Kohei Taniguchi, Nahoko Kato‐Kogoe, Shoichi Sakaguchi, Shota Nakamura, Yoshiro Imai, Takashi Nakano, Takaaki Ueno, Sang‐Woong Lee

**Affiliations:** ^1^ Department of General and Gastroenterological Surgery Osaka Medical and Pharmaceutical University Takatsuki Osaka Japan; ^2^ Department of General, Breast and Digestive Surgery Otsu City Hospital Otsu Shiga Japan; ^3^ Center for Medical Research & Development, Division of Translational Research Osaka Medical and Pharmaceutical University Takatsuki Osaka Japan; ^4^ Department of Dentistry and Oral Surgery, Faculty of Medicine Osaka Medical and Pharmaceutical University Takatsuki Osaka Japan; ^5^ Department of Microbiology and Infection Control, Faculty of Medicine Osaka Medical and Pharmaceutical University Takatsuki Osaka Japan; ^6^ Department of Infection Metagenomics, Genome Information Research Center, Research Institute for Microbial Diseases Osaka University Suita Japan

**Keywords:** 16S rRNA sequencing, gut microbiota, rectal cancer, stoma, stoma closure

## Abstract

**Aim:**

To investigate the impact of temporary stoma creation and its subsequent closure on gut microbiota composition and diversity in patients undergoing rectal cancer surgery.

**Methods:**

Nineteen patients with primary rectal cancer who underwent curative surgery were enrolled and divided into two groups: stoma (*n* = 10, all underwent temporary ileostomy) and non‐stoma (*n* = 9). Fecal samples were collected preoperatively and 6 months postoperatively. Gut microbiota composition was analyzed using 16S rRNA gene sequencing. Alpha diversity (observed operational taxonomic units and Shannon index) and beta diversity (UniFrac distances) were compared between time points. Taxonomic shift was identified using Linear discriminant analysis Effect Size (LEfSe).

**Results:**

In the stoma group, alpha diversity significantly decreased after surgery (*p* = 0.049), and beta diversity analyses revealed significant changes in microbial composition (PERMANOVA; unweighted *p* = 0.026; weighted *p* = 0.046). LEfSe analysis identified an increased abundance of potentially pathogenic genera (e.g., *Enterococcus* and *Eggerthella*) and a decreased abundance of short‐chain fatty acid‐producing genera (e.g., *Megamonas* and *Anaerostipes*). These changes persisted for at least 6 months after stoma closure. In contrast, the non‐stoma group showed no significant alterations in microbial diversity or composition over time.

**Conclusion:**

Temporary stoma creation in rectal cancer surgery induces persistent alterations in gut microbiota; these alterations are characterized by reduced diversity and a shift toward a dysbiotic profile with increased pathogenic and decreased beneficial taxa. These findings highlight the potential need for microbiota‐targeted strategies to mitigate long‐term dysbiosis in patients undergoing stoma‐related procedures.

## Graphical abstract

We investigated the impact of temporary stoma creation and closure on gut microbiota diversity in rectal cancer patients. 16S rRNA sequencing revealed that microbial diversity significantly decreased postoperatively in the stoma group but remained stable in non‐stoma patients.

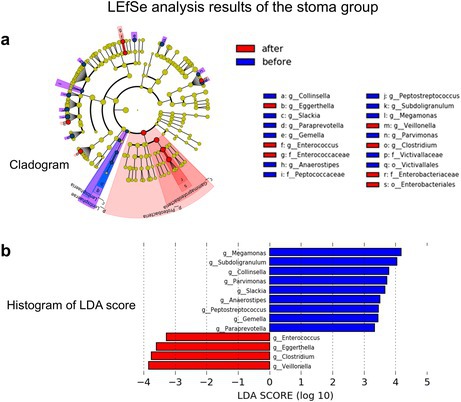

**Abbreviations**
16S rRNA: 16S ribosomal RNA
ISR: inter‐sphincteric resection
LAR: low anterior resection
LEfSe: linear discriminant analysis effect size
OTU: operational taxonomic unit
uLAR: ultra‐low anterior resection

## 1 Introduction

Advances in next‐generation sequencing technology have enabled rapid and comprehensive analysis of the intestinal microbiota, facilitating numerous studies on the physiological role of the human gut microbiota and its association with various cancers [1, 2].

Several studies have examined the relationship between colorectal cancer and gut microbiota. *Fusobacterium nucleatum* , colibactin‐producing pathogenic *Escherichia coli* , and toxin‐producing *Bacteroides fragilis* have been reported to be involved in the progression of colorectal cancer [3, 4].

The effect of colorectal cancer surgery on gut microbiota has also been investigated. Some studies have reported a reduction in gut microbiota after surgery compared with preoperative levels [5, 6]. Furthermore, some findings suggest a potential association between gut microbiota and surgical complications [7, 8]. Notably, in anastomotic leakage—the most concerning complication—an increase in *Lachnospiraceae* and *Bacteroidaceae* has been observed in affected leakage [9]. In addition, *Enterococcus faecalis* may contribute to collagen degradation, thereby inducing anastomotic leakage [10].

In colorectal cancer, particularly rectal cancer, a temporary (covering) stoma is often required. A temporary stoma provides clinical benefits such as promoting surgical site healing and reducing the risk of anastomotic leakage. However, stoma creation is associated with complications, including dermatitis, dehydration, reduced quality of life, and psychological distress, which present clinical challenges [11, 12]. Furthermore, some studies have reported that stoma closure may be associated with persistent or even worsened bowel dysfunction, including the development or exacerbation of low anterior resection syndrome (LARS). This highlights the need for careful patient selection and follow‐up [12].

In patients with a stoma, stool does not pass through the anus, resulting in a non‐physiological state. A longer transit time through the descending colon is associated with increased intestinal microbiota diversity [13]. However, this process is absent in patients with a stoma, potentially leading to alterations in gut microbiota composition. Several studies have reported reduced microbial diversity in patients with a stoma [14, 15, 16].

Despite these findings, the effect of stoma closure on gut microbiota remains unexplored. Stoma closure restores stool flow to a more physiological state, allowing the digestive tract to follow the same route as in cases where a stoma was not created during curative rectal cancer surgery. However, even if temporary, stoma creation and maintenance may induce lasting alterations in gut microbiota. Investigating the effects of stoma creation and closure on gut microbiota is essential for optimizing surgical strategies and minimizing associated complications.

In this study, we compared the gut microbiota of patients with rectal cancer before and after stoma closure and examined the impact of stoma closure on microbial composition and diversity. Additionally, by comparing these changes with those in patients with rectal cancer who underwent surgery without stoma creation, we aimed to clarify the differential effects of these surgical interventions on gut microbiota.

## 2 Methods

### 2.1 Patients

This study included 19 patients diagnosed with primary rectal cancer who underwent radical surgery following the Japanese guidelines for rectal cancer treatment [17]. Surgeries were performed at the Department of General and Gastroenterological Surgery, Osaka Medical and Pharmaceutical University Hospital, Takatsuki, Japan, between May 2018 and December 2019. All patients received routine pretreatment with oral laxatives. Among these patients, 10 underwent temporary ileostomy creation during surgery (hereafter referred to as the stoma group), while the remaining nine did not (non‐stoma group).

At our hospital, rectal cancer surgery is performed using robot‐assisted or laparoscopic approaches. Surgical procedures included low anterior, ultra‐low anterior, and intersphincteric resections [18]. The decision to create an ileostomy or not was ultimately made by the surgeon during surgery, after taking into consideration various factors such as the position of the lower edge of the tumor, tension at the anastomosis site, the size of the pelvic cavity, and the presence or absence of comorbidities. The ileostomy was closed in all 10 cases in the stoma group. The median time from the initial surgery to colostomy closure was 182 days, with a range of 94–371 days. Although the standard interval before closure was 6 months, closure was performed earlier or later in some cases.

Patients were excluded if they had macroscopic residual disease at the time of surgery, received preoperative chemotherapy or radiotherapy, or underwent simultaneous resection of other organs for malignancies. Additional exclusion criteria included immunosuppressant or antibiotic use within 3 months prior to specimen collection, ongoing infections, autoimmune diseases, renal or liver failure, or active treatment for malignant tumors. Standard bowel preparation with oral laxatives was performed for all patients, regardless of the surgical procedure. Patient characteristics, including sex, age, cancer stage, and surgical approach, are summarized in Table 1. Pathological staging was defined according to the Japanese classification of colorectal cancer [19]. All patients received BIO‐THREE (a probiotic tablet manufactured in Japan) at a dose of three tablets daily, starting immediately after surgery. Each tablet contained *Clostridium butyricum* TO‐A, *Bacillus subtilis* TO‐A (1 × 10^5^–10^8^ CFU), and *Enterococcus faecium* T‐110 (2 × 10^5^–4 × 10^8^ CFU). At the time of stool collection, 12 of 19 patients had discontinued the probiotic, whereas seven continued its use.

**TABLE 1:** Characteristics of patients with stoma and non‐stoma group.

	Stoma group (*n* = 10)	Non‐stoma group (*n* = 9)
Sex (M/F)	(6/4)	(5/4)
Age (years)	72.1 (51–82)	63.9 (51–79)
BMI	21.3 (17.3–24.9)	22.7 (09.5–24.9)
Stage (I/II/III)	(7/3/0)	(8/0/1)
Surgical approach (open/laparoscopic/robot)	(0/7/3)	(0/7/2)
Surgical procedure (LAR/uLAR/ISR)	(8/0/2)	(5/1/3)
BIOTHREE (+/−)	(3/7)	(4/5)
Median period until sampling after surgery (range)	230.5 (182–282)	212 (182–293)
Median period until stoma closure after radical surgery (range)	182 (94–371)	

Abbreviations: BIO‐THREE, aprobiotic mixture ( *Bacillus subtilis* , *Enterococcus faecium* , and *Clostridium butyricum* ); BMI, body mass index; ISR, intersphincteric resection; LAR, low anterior resection; uLAR, ultra‐low anterior resection.

This study was approved by the Institutional Review Board of Osaka Medical and Pharmaceutical University (approval number: 2145; approval date: 8 May 2017) and conducted in accordance with the Declaration of Helsinki. Written informed consent was obtained from all participants.

### 2.2 Sample Collection

**FIGURE 1:**
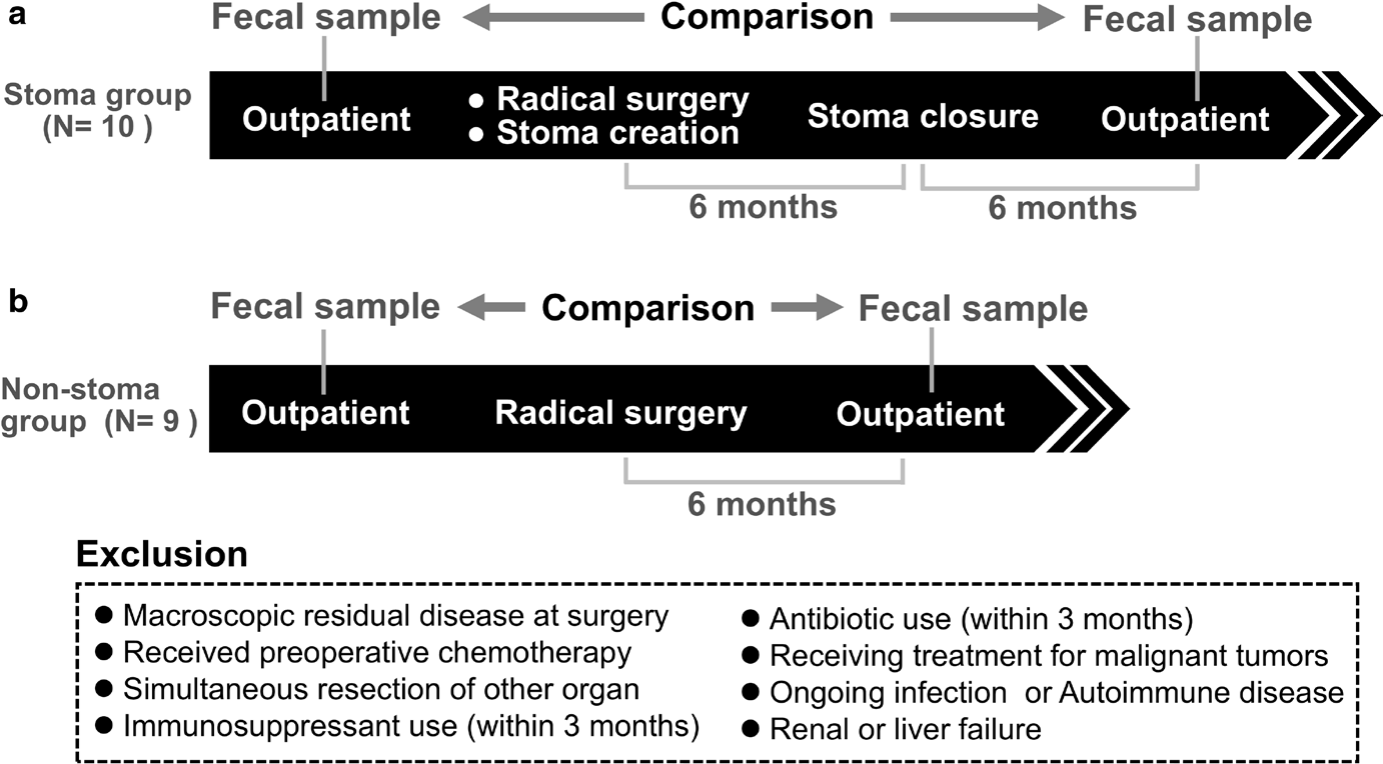
Study design and fecal sample collection timeline for stoma and non‐stoma groups. (a) Stoma group: Fecal samples were collected from patients undergoing radical rectal cancer surgery with temporary ileostomy creation (stoma) at two time points—before the initial surgery and at least 6 months after stoma closure. (b) Non‐stoma group: Fecal samples were collected from patients undergoing radical rectal cancer surgery without stoma creation at two time points—before the initial surgery and at least 6 months post‐surgery.

Fecal samples were collected from each patient at two time points: before the initial surgery and at least 6 months after the final surgery, in accordance with previous reports indicating that the intestinal flora undergoes temporary changes after surgery and reaches a stable state around 6 months postoperatively [7]. In the stoma group, samples were collected before the initial surgery and at least 6 months after stoma closure. In the non‐stoma group, samples were collected before surgery and at least 6 months after rectal cancer surgery. Gut microbiota diversity was compared between these time points (Figure 1), specifically examining diversity changes in patients with and without stomas. The median period from surgery to stool sample collection was 230.5 days (range, 182–282 days) in the stoma group and 212.0 days (range, 182–293 days) in the non‐stoma group.

### 2.3 DNA Extraction, 16S rRNA Sequencing, and Taxonomic Classification

Fecal samples were collected using the Mykinso fecal collection kit (Cykinso, Tokyo, Japan) containing guanidine thiocyanate solution, transported at room temperature, and stored at 4°C. DNA was extracted using an automated DNA extraction system (GENE PREP STAR PI‐480, Kurabo Industries Ltd., Osaka, Japan) according to the manufacturer's protocol and prior studies [16, 20, 21, 22]. The V1–V2 region of the 16S rRNA gene was amplified using the forward primer 16S_27Fmod (TCG GCA GCG TCA GAT GTG TAT AAG AGA CAG AGR GTT TGA TYM TGG CTC AG) and the reverse primer 16S_338R (GTC TCG TGG GCT CGG AGA TGT GTA TAA GAG ACA GTG CTG CCT CCC GTA GGA GT). The library was prepared following the 16S rRNA Sequencing Library Preparation Protocol (Illumina, San Diego, CA, USA) and sequenced for 500 cycles using the MiSeq Reagent Kit v2 (Illumina).

### 2.4 Statistical Analysis

Statistical analyses were performed as described previously [21, 22]. All analyses were conducted using JMP Pro 15.1.0 (Version 15, SAS Institute, Cary, North Carolina, USA). Categorical variables were reported as frequency counts and compared using Fisher's exact test or the Wilcoxon rank‐sum test, whereas continuous variables were analyzed using Student's *t*‐test. Statistical significance was set at *p* < 0.05.

Intra‐participant alpha diversity of bacterial communities was assessed using observed operational taxonomic units (OTUs) and the Shannon index [23]. Group comparisons were performed using the Kruskal–Wallis test, with significance set at *p* < 0.05. Interparticipant beta diversity was evaluated using unweighted and weighted UniFrac distance metrics [23]. Principal coordinate analysis was used to visualize microbiome structure differences, and significance was assessed with permutational multivariate analysis of variance (PERMANOVA) using QIIME2 [24]. The Greengenes reference database (version 13.8) was used for taxonomic classification.

The Linear discriminant analysis Effect Size (LEfSe) algorithm [16] was used to identify the abundance of different bacteria before and after surgery in the stoma group. The relative abundance of specific bacterial genera was compared between the pre‐ and postoperative groups using the Wilcoxon signed rank test, with statistical significance set at *p* < 0.05. The LEfSe algorithm was applied only to the stoma group, which showed significant changes in alpha and beta diversities.

## 3 Result

### 3.1 Patients' Characteristics

The study included 10 patients in the stoma group who underwent radical surgery with stoma creation for rectal cancer, followed by stoma closure, and nine patients in the non‐stoma group who underwent radical surgery without stoma creation (Figure 1). The mean ages of the stoma and non‐stoma groups were 72.1 and 65.0 years, respectively, with male‐to‐female ratios of 6:4 and 6:5, respectively. None of the patients received chemotherapy. There were no significant differences in baseline characteristics between the groups. Patient characteristics are summarized in Table 1.

### 3.2 Microbiota Composition Before and After Surgery in the Stoma and Non‐Stoma Groups

**FIGURE 2:**
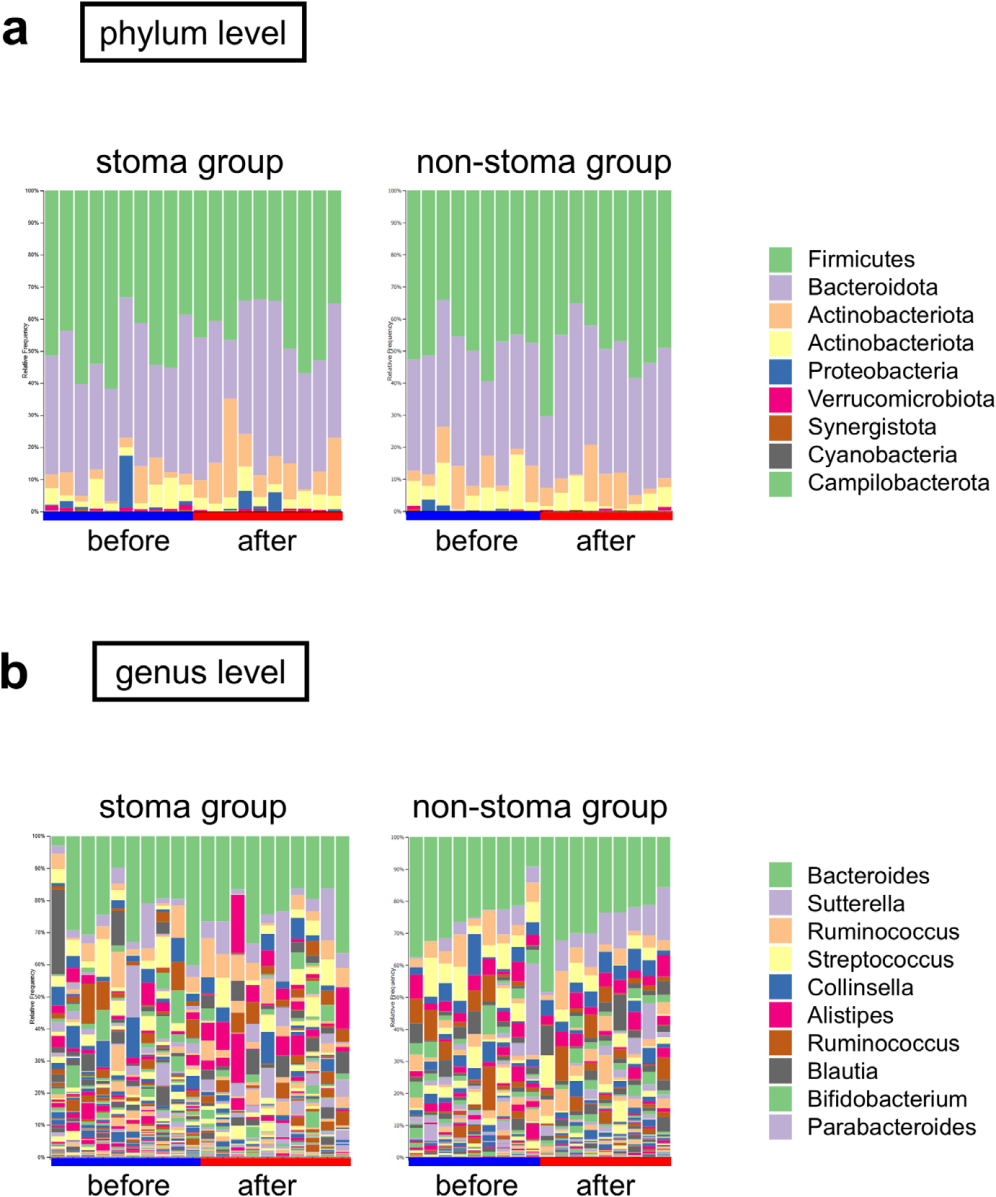
Taxonomic composition of gut microbiota in stoma and non‐stoma groups before and after surgery. (a) Composition at the phylum level. (b) Composition at the genus level.

As shown in Figure 2, the taxonomic compositions of the gut microbiota in the two groups before and after surgery are illustrated. In the stoma group, bacteria with a relative abundance of ≥ 0.1% were classified into 7 phyla, 12 classes, 30 orders, 30 families, and 152 genera before and after surgery. Similarly, in the non‐stoma group, these bacteria were classified into 7 phyla, 11 classes, 25 orders, 42 families, and 106 genera.

At the phylum level, the dominant bacteria in the stoma group (> 5% of the total sequences) included *Firmicutes*, *Bacteroidetes*, and *Proteobacteria*, which accounted for 91.99% and 94.03% of the gut microbiota before and after surgery, respectively. In the non‐stoma group, the dominant phyla were *Firmicutes*, *Bacteroidetes*, *Proteobacteria*, and *Actinobacteria*, constituting 98.7% and 99.4% of the gut microbiota before and after surgery, respectively (Figure 2a).

A total of 225 genera were identified in the stoma group before surgery, 47 of which were absent postoperatively. Conversely, 212 genera were identified postoperatively, 34 of which were absent preoperatively, with 178 genera common to both time points. In the non‐stoma group, 210 genera were identified preoperatively, 29 of which were absent postoperatively, whereas 214 genera were identified postoperatively, 33 of which were absent preoperatively, with 181 genera common to both time points (Figure 2b).

The relative abundances of the three bacterial strains contained in BIO‐THREE were not consistently elevated in patients who continued its use. Detailed results are shown in Table S1.

### 3.3 Changes in Gut Microbiota Before and After Surgery in the Stoma and Non‐Stoma Groups

In the present study, we evaluated changes in gut microbiota diversity by comparing pre‐ and postoperative samples within each group—the stoma group and the non‐stoma group. Comparisons were limited to the detection of significant within‐group changes. Direct statistical comparisons between the stoma and non‐stoma groups were not performed.

**FIGURE 3:**
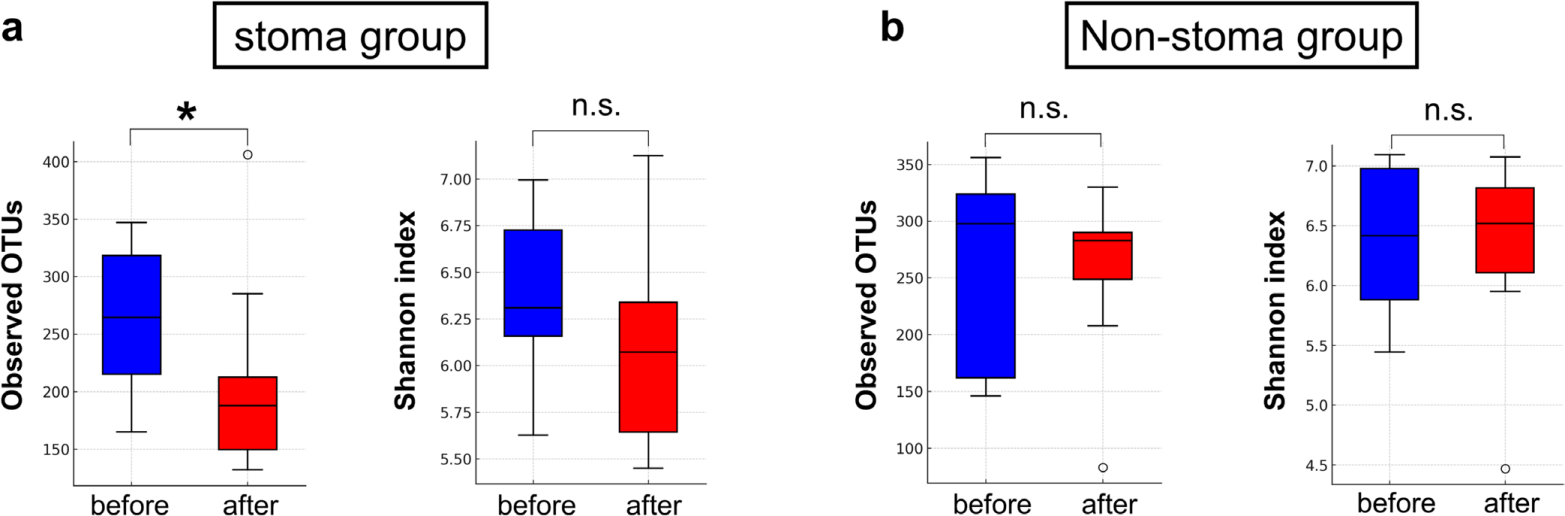
Alpha diversity of gut microbiota in stoma and non‐stoma groups. (a) Alpha diversity in the stoma group. (b) Alpha diversity in the non‐stoma group. Alpha diversity was assessed using the observed operational taxonomic unit (OTU) and Shannon indices. Measurements were conducted at two time points: Before surgery (blue) and after surgery (red). *p < 0.05. Comparisons between groups were performed using the Kruskal–Wallis test. n.s., no significant difference.

As shown in Figure 3a, the richness (alpha diversity) of the gut microbiota in the stoma group significantly decreased postoperatively, relative to that before surgery (observed OTUs, *p* = 0.049). In contrast, the non‐stoma group showed no significant difference in richness (observed OTUs) between the pre‐ and postoperative periods (Figure 3b). The Shannon diversity index, which accounts for both richness and evenness of microbial communities, showed no statistically significant differences in either group, although a slight decrease was observed in the stoma group (Figure 3a) while the index remained stable in the non‐stoma group (Figure 3b).

**FIGURE 4:**
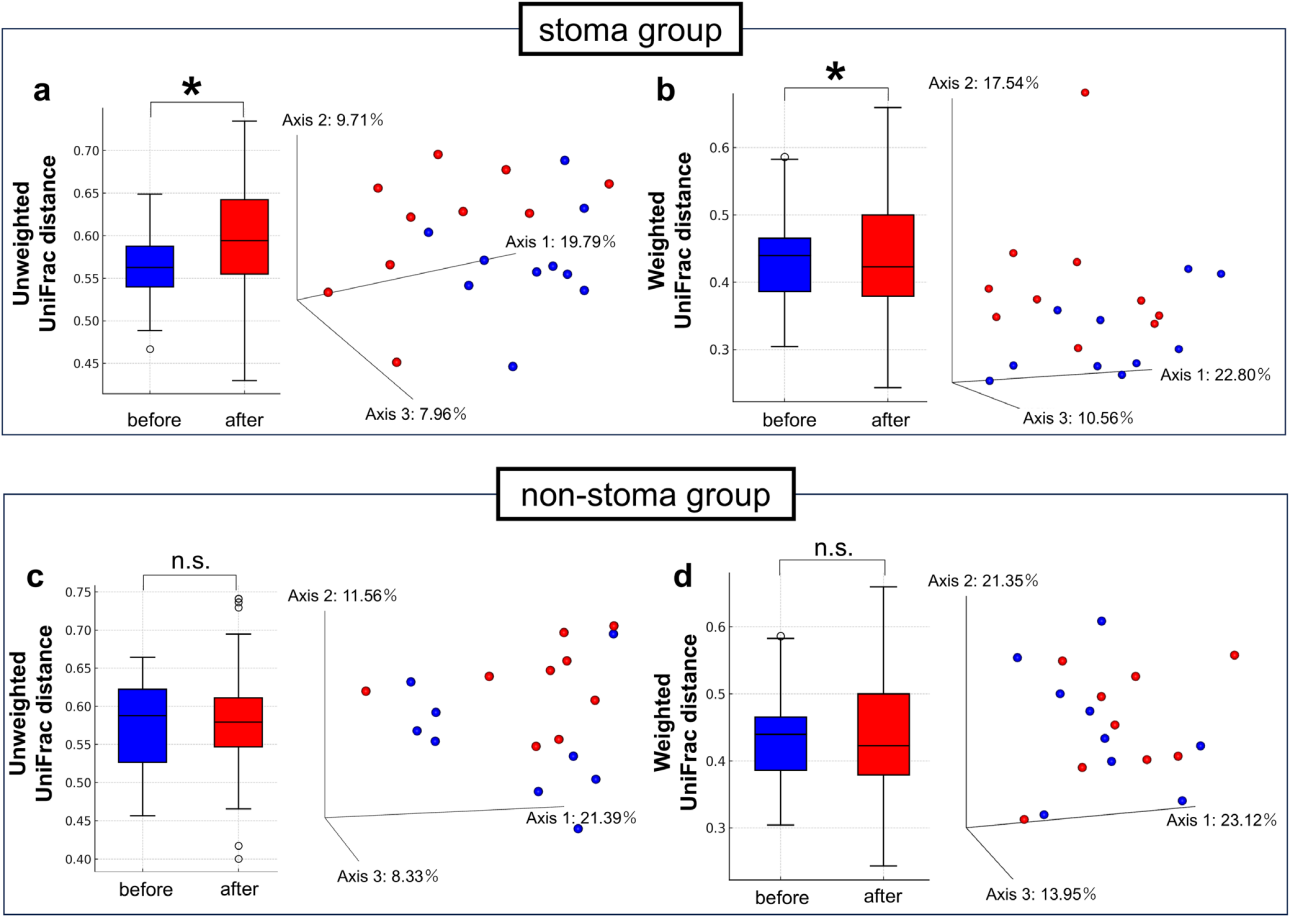
Changes in beta diversity of gut microbiota before and after surgery in the stoma and non‐stoma groups. (a) Unweighted UniFrac distances in the stoma group. (b) Weighted UniFrac distances in the stoma group. (c) Unweighted UniFrac distances in the non‐stoma group. (d) Weighted UniFrac distances in the non‐stoma group. Principal coordinate analysis (PCoA) plots show samples from patients at two time points: Before (blue) and after (red) surgery. Box plots represent UniFrac distances comparing these two time points, with blue columns representing before surgery and red columns representing after surgery. *p < 0.05. Group comparisons were performed using permutational multivariate analysis of variance (PERMANOVA) with 999 permutations. n.s., no significant difference.

Principal coordinate analysis based on unweighted (Figure 4a) and weighted (Figure 4b) UniFrac distances in the stoma group revealed differences in gut microbiota composition between preoperative and postoperative samples in three‐dimensional space. These compositional differences were further validated using PERMANOVA (unweighted, *p* = 0.026; weighted, *p* = 0.046). In contrast, similar analyses in the non‐stoma group did not show significant compositional differences between preoperative and postoperative samples (unweighted, *p* = 0.16; Figure 4c; weighted, *p* = 0.47; Figure 4d).

### 3.4 Bacteria With Differential Abundances Before and After Surgery in the Stoma Group

**FIGURE 5:**
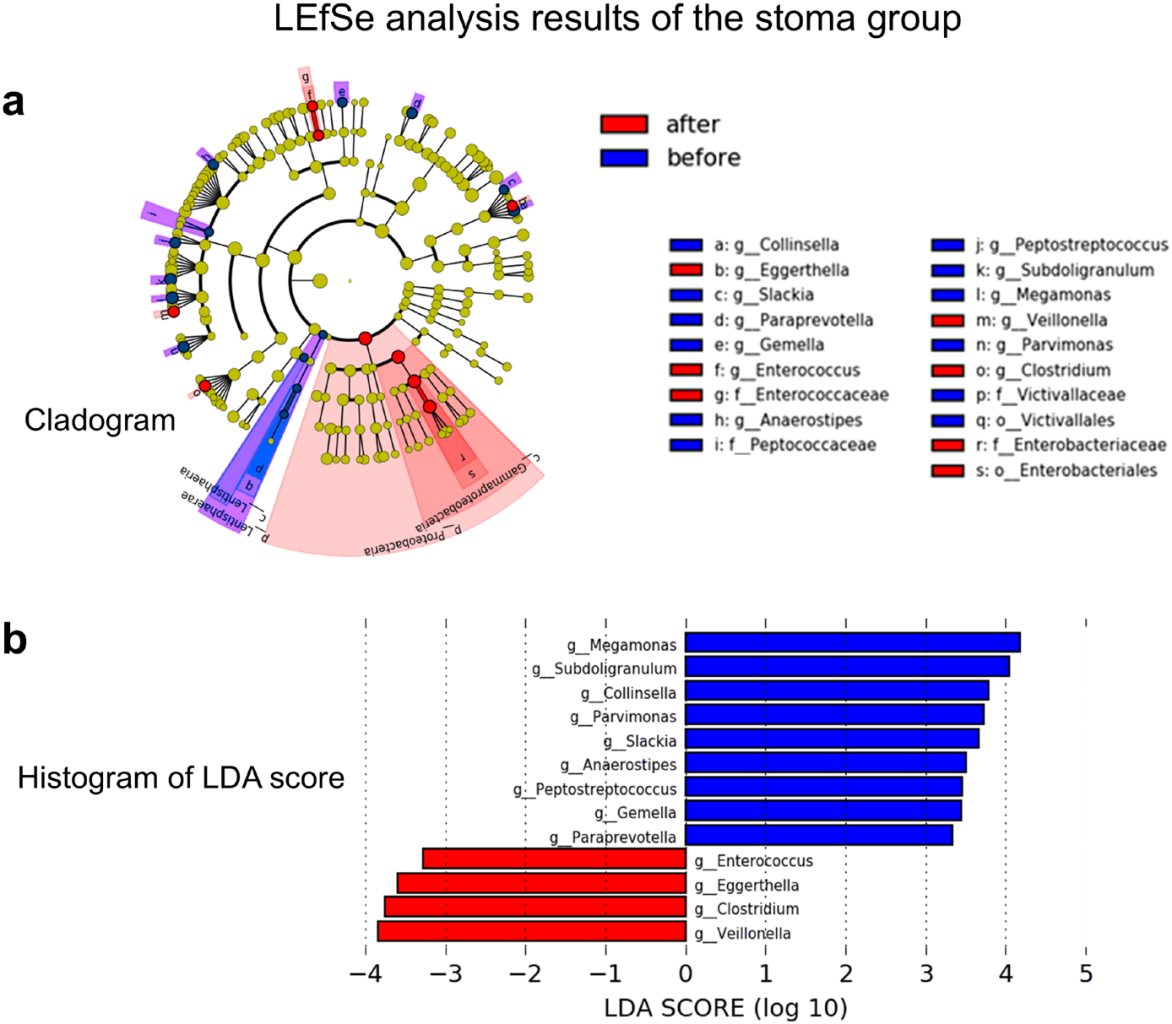
Differentially abundant bacterial genera in the stoma group identified by linear discriminant analysis effect size (LEfSe). (a) A phylogenetic tree displaying differentially abundant bacterial taxa. Each layer represents a taxonomic rank, with the central point representing the root (bacteria) and subsequent rings indicating descending taxonomic levels (p, phylum; c, class; o, order; f, family; g, genus). Taxa enriched in the pre‐surgery group are shown in blue, while those enriched in the post‐surgery group are shown in red. The diameter of each circle indicates the relative abundance of the taxon. (b) A histogram of linear discriminant analysis (LDA) scores for differentially abundant taxa between the pre‐ and post‐surgery groups, with LDA scores ≥ 2.0. Red bars indicate taxa significantly increased in the post‐surgery group, and blue bars indicate taxa significantly increased in the pre‐surgery group.

LEfSe analysis was performed for the stoma group, which showed changes in both alpha diversity and beta diversity. Figure 5a presents a cladogram showing taxa with significant differences before and after surgery in the stoma group. The circular tree highlights taxonomic ranks from phylum to genus. Figure 5b shows linear discriminant analysis scores for genera with significant changes: *Enterococcus*, *Eggerthella*, *Clostridium*, and *Veillonella* increased postoperatively, while *Megamonas*, *Subdoligranulum*, *Collinsella*, *Parvimonas*, *Slackia*, *Anaerostipes*, *Peptostreptococcus*, *Gemella*, and *Paraprevotella* decreased.

## 4 Discussion

In this study, we investigated the gut microbiota in stool samples from patients who underwent radical surgery with stoma creation for rectal cancer, followed by stoma closure, using 16S rRNA sequencing. To our knowledge, this is the first study to compare gut microbiota composition in stool samples before and after stoma creation and closure.

Our findings demonstrate a significant decrease in gut microbiota diversity before and after surgery in patients who underwent temporary stoma creation by examining alpha diversity using observed OTUs. We also observed significant changes in the composition of the gut microbiota by examining beta diversity. Furthermore, a comparison of bacterial composition before and after surgery revealed a shift toward a microbial profile that may be detrimental to patients. Notably, these alterations in gut microbiota composition and diversity persisted even 6 months after stoma closure, indicating that the temporary interruption of fecal flow due to stoma creation has long‐lasting effects on the intestinal microbial environment. The observed OTUs reflect species richness, while the Shannon index considers both richness and evenness. The unchanged Shannon index suggested that, despite a reduction in the number of taxa, the overall microbial balance was maintained.

In the non‐stoma group, no significant changes in gut microbiota composition or diversity were observed before and after surgery. This finding contrasts with previous studies reporting a postoperative decrease in alpha diversity in patients with rectal cancer compared with both their preoperative microbiota and that of healthy individuals. One possible explanation for this discrepancy is the small sample size of the non‐stoma group in the present study (*n* = 9), which may have limited statistical power to detect significant differences. Moreover, only one prior study specifically focused on rectal cancer, and in that study, 6 of 10 patients had stage ≥ III cancer. In contrast, in the present non‐stoma group, only 1 of 9 patients had stage III disease. This difference in disease stage distribution suggests that the previous study may have included more advanced cases, potentially influencing gut microbiota composition. Therefore, the inconsistency between studies may be attributable to differences in disease severity and potential selection bias.

As described in the Section 2, we observed significant changes in both alpha and beta diversities between pre‐ and postoperative stool samples in the stoma group. Accordingly, we performed LEfSe analysis at the genus level to identify bacterial taxa that significantly increased or decreased after surgery in this group.

Although stool samples from the ileostomy itself were not collected in this study, a previous report analyzed microbial changes by comparing stool samples collected from the ileostomy with those obtained before stoma creation [15]. In the present study, *Enterococcus*, a facultative anaerobe, was found to increase after surgery in the stoma group, a finding consistent with the results of a study by Sakai et al. [15], which demonstrated changes in the microbiota composition between pre and post‐stoma samples collected directly from the ileostomy. A previous study also showed that stool samples from stoma patients undergoing colorectal cancer surgery exhibit reduced microbial diversity, characterized by a decrease in obligate anaerobes and an increase in facultative anaerobes and aerobes (e.g., *Enterococcus*, *Pseudomonas*, and *Acinetobacter*) [15]. These changes in ileostomy‐associated microbiota are likely due to the lack of colonic passage. It is well established that in the colon, the partial pressure of oxygen decreases from the proximal to the distal segments, leading to a corresponding increase in the abundance of obligate anaerobes [25, 26]. These findings align with the physiological changes induced by an ileostomy, which bypasses the colon and exposes the microbiota to a relatively oxygenated environment. This environment favors the growth of facultative anaerobes and aerobes. The observed increase in *Enterococcus*, a facultative anaerobe, between pre‐ and postoperative samples in the stoma group in this study supports the findings of the previous study [15] on ileostomy‐associated microbiota; this suggests that such microbiota alterations may persist even after stoma closure.

In this study, LEfSe analysis revealed specific genera with significantly different relative abundances in the stoma group before and after surgery. *Enterococcus*, *Eggerthella*, *Clostridium*, and *Veillonella* include species with pathogenic potential, depending on the strain [27, 28]. In this study, the abundance of these bacteria increased postoperatively in the stoma group. Conversely, among the bacteria that decreased after surgery in the stoma group, Megamonas, Subdoligranulum, Collinsella, Anaerostipes, Paraprevotella, and Peptostreptococcus are short‐chain fatty acid (SCFA)‐producing bacteria. A reduction in SCFA‐producing bacteria is generally associated with disruption of intestinal pH and microbial balance, potentially promoting the overgrowth of harmful bacteria [29, 30]. In summary, the stoma group exhibited decreased microbial diversity, a reduction in SCFA‐producing bacteria, and an increase in infection‐associated bacteria, all of which may pose a risk to patient health.

In this exploratory study, we aimed to identify patterns of microbiota alterations associated with diverting ileostomy. In rectal cancer surgery, even when a temporary stoma is created and subsequently closed, the final route of fecal passage becomes anatomically identical to that of patients without a stoma (non‐stoma group). Notably, patients who had undergone temporary ileostomy exhibited a sustained reduction in gut microbial diversity even after stoma closure. This was accompanied by an increased abundance of potentially pathogenic genera such as *Enterococcus* and *Clostridium* and a decrease in beneficial SCFA‐producing bacteria. These alterations may contribute to postoperative bowel dysfunction or infectious complications; however, clinical outcomes were not assessed in this cohort. Further studies incorporating clinical outcome measures are warranted to clarify the clinical implications of these findings.

In this study, patients received BIO‐THREE at a dosage of three tablets per day after surgery, and seven were still taking it at sample collection. Probiotics may influence gut microbiota composition [31]; therefore, the conditions for probiotic administration should ideally be standardized to minimize potential confounding. However, by analyzing the bacterial composition in individual stool samples, we investigated the three strains of bacteria contained in BIO‐THREE and found that they were not consistently elevated in patients who continued to take the probiotic. Thus, we considered the impact to be minimal. Because the number of cases was small and further subdivision of groups was not statistically possible, we did not stratify patients by the amount of probiotic intake. Previous studies have reported that BIO‐THREE administration may increase SCFA‐producing bacteria and improve clinical symptoms [32, 33]. However, the dose used in this study was lower than in previous studies, which may explain why none of the probiotic strains significantly influenced gut microbiota composition. It may be necessary to increase the dosage to elicit the beneficial effects of these probiotics.

This study has several limitations. The small sample size of 19 participants limits the generalizability of the findings, particularly given the individual variability in gut microbiota, highlighting the need for larger studies. According to this study's results, we calculated that at least 15 patients per group would be required to achieve a statistical power of 80%, using the observed change in diversity (alpha diversity) before and after surgery as the standard. In this study, clinical symptoms such as postoperative bowel function were not recorded; therefore, the relationship between the severity of LARS and the gut microbiota could not be evaluated. Seven patients received probiotics postoperatively; this may have potentially affected the gut microbiota. Moreover, inconsistent probiotic administration complicates the assessment of its effects. Additionally, this study examined microbiota changes only at 6 months post‐surgery, leaving long‐term effects unaddressed. Although this study focused on the effects of stoma creation and closure on gut microbiota, other factors such as diet and medication during recovery were not fully explored.

Further studies are needed to investigate the clinical consequences of gut microbiota changes, particularly in relation to postoperative infections, inflammation, bowel function, stool frequency, and quality of life. In particular, it seems necessary to investigate the association between microbiota changes and the severity of postoperative defecation disorders and LARS. Quantitative evaluation of postoperative bowel function using the Wexner Incontinence Score and Fecal Incontinence Quality of Life Scale may be necessary to explore the relationship between changes in gut microbiota and clinical symptoms. Longitudinal studies could provide more insight into the long‐term effects of stoma‐related surgeries on gut microbiota and their impact on rectal cancer survival rates.

## 5 Conclusions

This study demonstrated that stoma creation resulted in significant alterations in gut microbiota diversity and composition, which persisted for at least 6 months after stoma closure. Specifically, the stoma group exhibited a sustained decrease in alpha diversity and notable postoperative shifts in beta diversity, whereas the non‐stoma group showed no significant changes in either diversity measure.

## Author Contributions

**Yusuke Suzuki:** conceptualization, writing – original draft, data curation. **Wataru Osumi:** conceptualization, data curation. **Kohei Taniguchi:** conceptualization, writing – review and editing, project administration. **Nahoko Kato‐Kogoe:** conceptualization, data curation, project administration, writing – review and editing. **Shoichi Sakaguchi:** data curation, formal analysis, writing – review and editing, project administration. **Shota Nakamura:** formal analysis. **Yoshiro Imai:** data curation. **Takashi Nakano:** project administration, formal analysis. **Takaaki Ueno:** project administration. **Sang‐Woong Lee:** project administration.

## Ethics Statement

This study was approved by the Institutional Review Board of Osaka Medical and Pharmaceutical University (acceptance number: 2145; approval date: 8/5/2017) and was conducted in accordance with the Declaration of Helsinki.

## Consent

Blanket consent was obtained from the patients for the publication.

## Conflicts of Interest

The authors declare no conflicts of interest.

## Supporting information


**Table S1:** Percentage of BIO‐THREE constituent bacteria in each sample before and after surgery for each case.

## Data Availability

The datasets used and/or analyzed in this study are available upon request. If someone wants to request data from this study, they can request it from the corresponding author.
