# Supplementary material for: Comparison of Pre‐ and Postoperative Gut Microbiota Diversity in Patients With Rectal Cancer Undergoing Stoma Creation and Closure
Source: Ann Gastroenterol Surg. 2025 Sep 16;10(2):492–501. doi: 10.1002/ags3.70094 (PMC12962007; doi:10.1002/ags3.70094)
Supplement: Supplementary file 1 — Table S1: Percentage of BIO‐THREE constituent bacteria in each sample before and after surgery for each case. [file AGS3-10-492-s001.docx]

Supplementary table 1

| Cases | stoma | gender | Continued administration of  BIO-THREE® | Clostridium butyricum | | Bacillus subtilis | | Enterococcus faecium | |
| --- | --- | --- | --- | --- | --- | --- | --- | --- | --- |
|  |  |  |  | before | after | before | after | before | after |
| 1 | - | F | - | 0 | 0 | 0 | 0 | 0 | 0 |
| 2 | - | F | - | 0 | 0 | 0 | 0 | 0 | 0 |
| 3 | - | M | - | 0 | 0 | 0 | 0 | 0 | 0 |
| 4 | - | F | - | 0 | 0 | 0 | 0 | 0 | 0 |
| 5 | - | M | - | 0 | 0 | 0 | 0 | 0 | 0 |
| 6 | - | M | + | 0 | 0 | 0 | 0 | 0 | 0 |
| 7 | - | M | + | 0 | 0 | 0 | 0 | 0 | 0 |
| 8 | - | F | + | 0 | 0 | 0 | 0 | 0 | 0 |
| 9 | - | M | + | 0 | 0 | 0 | 0 | 0 | 0 |
| 10 | + | M | - | 0 | 0 | 0 | 0 | 0.0188 | 0 |
| 11 | + | M | - | 0 | 0 | 0 | 0 | 0.0087 | 0 |
| 12 | + | F | - | 0 | 0 | 0 | 0 | 0 | 0 |
| 13 | + | M | - | 0 | 0 | 0 | 0 | 0 | 0.0238 |
| 14 | + | M | - | 0 | 0 | 0 | 0 | 0.0086 | 0.0098 |
| 15 | + | F | - | 0 | 0.0477 | 0 | 0 | 0.0536 | 0 |
| 16 | + | M | - | 0 | 0 | 0 | 0 | 0.0127 | 0 |
| 17 | + | M | + | 0 | 0.0003 | 0 | 0 | 0 | 0 |
| 18 | + | F | + | 0 | 0 | 0 | 0 | 0 | 0 |
| 19 | + | F | + | 0 | 0 | 0 | 0 | 0 | 0.0307 |

Supplementary Table 1 shows the percentage of BIO-THREE® constituent bacteria in each sample before and after surgery for each case.
